# Reducing small intestinal permeability attenuates colitis in the IL10 gene-deficient mouse

**DOI:** 10.1136/gut.2008.150888

**Published:** 2008-10-01

**Authors:** M C Arrieta, K Madsen, J Doyle, J Meddings

**Affiliations:** 1 Division of Gastroenterology, Department of Medicine, University of Alberta, Edmonton, Canada; 2 Department of Laboratory Medicine and Pathology, Vernon Jubilee Hospital, Vernon, British Columbia, Canada

## Abstract

**Background:**

Defects in the small intestinal epithelial barrier have been associated with inflammatory bowel disease but their role in the causation of disease is still a matter of debate. In some models of disease increased permeability appears to be a very early event. The interleukin 10 (IL10) gene-deficient mouse spontaneously develops colitis after 12 weeks of age. These mice have been shown to have increased small intestinal permeability that appears early in life. Furthermore, the development of colitis is dependent upon luminal agents, as animals do not develop disease if raised under germ-free conditions.

**Aims:**

To determine if the elevated small bowel permeability can be prevented, and if by doing so colonic disease is prevented or attenuated.

**Methods:**

IL10 gene-deficient (IL10^−^/^−^) mice) were treated with AT-1001 (a zonulin peptide inhibitor), a small peptide previously demonstrated to reduce small intestinal permeability. Small intestinal permeability was measured, in vivo, weekly from 4 to 17 weeks of age. Colonic disease was assessed at 8 weeks in Ussing chambers, and at 17 weeks of age inflammatory cytokines and myeloperoxidase were measured in the colon. Colonic permeability and histology were also endpoints.

**Results:**

Treated animals showed a marked reduction in small intestinal permeability. Average area under the lactulose/mannitol time curve: 5.36 (SE 0.08) in controls vs 3.97 (SE 0.07) in the high-dose AT-1001 group, p<0.05. At 8 weeks of age there was a significant reduction of colonic mucosal permeability and increased electrical resistance. By 17 weeks of age, secretion of tumour necrosis factor α (TNFα) from a colonic explant was significantly lower in the treated group (25.33 (SE 4.30) pg/mg vs 106.93 (SE 17.51) pg/ml in controls, p<0.01). All other markers also demonstrated a clear reduction of colitis in the treated animals. Additional experiments were performed which demonstrated that AT-1001 was functionally active only in the small intestine.

**Conclusions:**

This work suggests that increased intestinal permeability may be an important aetiological event in the development of colitis in IL10^−^/^−^ mice.

Increased intestinal permeability has been proposed as a cause of systemic disease for decades. In several autoimmune diseases such as diabetes, Crohn’s disease and coeliac disease, increased intestinal permeability has been recognised as an early feature of the disease. In Crohn’s disease, increased intestinal permeability has been described in non-inflamed portions of the gut, as well as in healthy first-degree relatives of patients, suggesting that this defect can occur independently of inflammation.^1^^–^5 However, whether this is an epiphenomenon, an early manifestation of disease or a critical step in disease pathogenesis is unknown and has been the subject of much debate.

Intestinal barrier function involves dynamic and complex structures located at the junctions between intestinal epithelial cells. These junctions open and close continually in response to physiological^6^^–^8 and pathogen-induced stimuli.^9^^–^10 One of several physiological pathways implicated in increased intestinal permeability is the zonulin pathway. This pathway was postulated following the observation of the interaction between the bacterial pathogen *Vibrio cholerae* and the intestinal epithelial barrier. One of the several toxins produced by this bacterium, the zonula occludens toxin (ZOT), increases intestinal permeability. This toxin and its mammalian homologue zonulin is thought to bind to an apical membrane receptor on the enterocyte.^11^ Activation of this pathway initiates a cascade of intracellular events that involve phosphorylation of tight junction proteins and opening of the paracellular space.^12^ Zonulin secretion has been reported to be upregulated in coeliac disease,^13^ type 1 diabetes^14^ and in dermatitis herpetiformis.^15^ It has been suggested that the resultant increase in small intestinal permeability observed in these conditions is secondary to zonulin secretion and may be important in the pathogenesis of these diseases.

A synthetic octapeptide corresponding to amino acids in the receptor-binding motif of zonulin has been synthesised as a zonulin inhibitor. This zonulin peptide inhibitor (AT-1001) competitively blocks the apical zonulin receptor and prevents the opening of tight junctions secondary to zonulin.^11^ This inhibition is believed to only occur in the small intestine as the ZOT/zonulin receptor has been found in the jejunum and distal ileum, but not in the colon.^16^ We elected to use this compound, given chronically in the water supply, to determine whether increased small intestinal permeability could be prevented in the interleukin 10 gene deficient (IL10^−^/^−^) mouse.

This mouse develops a patchy, chronic colitis similar to human Crohn’s disease.^17^ From the point of view of this study, the IL10^−^/^−^ mouse model of disease has two important features. First, these animals have been described as having increased small intestinal permeability from very early in life and well before the onset of disease.^18^ Second, disease development is dependent upon luminal factors; it does not occur in animals raised under germ-free conditions. These observations suggested that the colitis observed in these animals might develop as a consequence of abnormal small intestinal permeability with increased presentation of a luminal agent to the mucosal immune system. This is a hypothesis that we have previously proposed for several other autoimmune diseases.^19^

Perhaps the best evidence suggesting that increased intestinal permeability has an aetiological role in autoimmune disease comes from the BB rat model of diabetes. In these animals autoimmune type 1 diabetes develops spontaneously in animals fed a normal diet, but can be prevented with a hydrolysed diet. We have previously demonstrated that these animals have increased gastric and small intestinal permeability even when receiving a hydrolysed diet.^20^ Furthermore, the expression of diabetes in animals consuming a normal diet can also be prevented by abolishing the increase in intestinal permeability with the zonulin receptor antagonist AT-1001.^21^ These data strongly suggest that, at least in this animal model of autoimmune disease, dietary antigens can initiate disease through a mechanism that involves increased gastrointestinal permeability. It also provides support for the concept that there may be novel means of preventing some autoimmune diseases by targeting increases in gastrointestinal permeability.

Therefore the goals of this study were 2-fold. First, we wished to determine whether the previously reported leakiness of the small intestine could be prevented by treating IL10^−^/^−^ animals with a zonulin receptor inhibitor. Second, if we could prevent the increase in small intestinal permeability observed in these animals would this prevent or ameliorate the subsequent colitis?

## MATERIALS AND METHODS

### Animals

Homozygous IL10^−/−^ mice generated on a 129 Sv/Ev genetic background, and 129 Sv/Ev controls were housed under specific pathogen-free conditions until weaning (3 weeks), when they were moved to conventional animal housing. The mice were housed in high-efficiency particulate air (HEPA) filter cages and fed a standard mouse chow diet. These mice were bred and raised in the animal facility at the University of Alberta.

### Pharmacological therapy

IL10^−^/^− ^mice aged 3.5 weeks and 129 Sv/Ev controls were randomised into four groups (n = 10–13 for each group). Starting at 4 weeks of age, two groups of IL10^−^/^−^ mice were treated with AT-1001 in their drinking water, whereas one group of IL10^−^/^−^ mice and one group of 129 Sv/Ev mice did not receive the treatment. The drinking water of the two treatment groups was prepared daily by dissolving either 0.1 mg/ml (low dose) or 1.0 mg/ml (high dose) of AT-1001 in filtered distilled water. Placebo groups drank filtered distilled water prepared daily as well. Mice were treated until 17 weeks of age, when they were killed by cervical dislocation.

In separate experiments, 10–12-week-old IL10^−^/^−^ mice and 129 Sv/Ev mice were given the zonulin receptor agonist AT-1002 (1 mg/ml) or AT-1002 mixed with AT-1001 (1 mg/ml each) in the drinking water for 24 h. A control group was given bovine serum albumin (1 mg/ml). Mice were placed in metabolic cages during these 24 h for urine collection.

AT-1001 and AT-1002 were graciously provided by Alba Therapeutics (Baltimore, Maryland, USA) as a neat, powdered chemical.

### Ussing chamber assay

Mice were killed at 8 weeks of age by cervical dislocation and a segment of colon removed. The mucosa was mounted in Lucite chambers exposing mucosal and serosal surfaces to 10 ml of oxygenated Krebs buffer (in mmol/l: 115 NaCl, 8 KCl, 1.25 CaCl_2_, 1.2 MgCl_2_, 2 KH_2_PO_4_, 225 NaHCO_3_; pH 7.35). The buffers were maintained at 37°C by a heated water jacket and circulated by CO_2_/O_2_. Fructose (10 mmol/l) was added to the serosal and mucosa sides. For measurement of basal mannitol fluxes, 1 mmol/l of mannitol with 370 KBO_r_ of ^3^H was added to the mucosal side. The spontaneous transepithelial potential difference (PD) was determined, and the tissue was clamped at zero voltage by continuously introducing an appropriate short-circuit current with an automatic voltage clamp (DVC 1000 World Precision Instruments, Sarasota, Florida, USA). Tissue ion resistance (1/G), where G is conductance, was calculated from the potential difference and short-circuit current according to Ohm’s law.^22^

### In vivo permeability measurement

Once every week, all mice were housed in metabolic cages after a 4 h fast of food and water and immediately after a gavage of 0.2 ml of a sugar probe containing 100 mg of sucrose, 12 mg of lactulose, 8 mg of mannitol and 6 mg of sucralose. To compensate for the 4 h without zonulin inhibitor treatment, experimental mice were fed 30 µl, by pipette, of a solution containing either 0.083 mg (low dose) or 0.83 mg (high dose) of AT-1001, 45 min before placing them in metabolic cages. Placebo groups received 30 μl of filtered distilled water. After the collection of urine the animals were placed in their respective cages, and provided with food and water.

Urine from each animal was collected for 22 h in collection tubes, treated with 100 µl of a 10% thymol solution (1.0 g/10 ml isopropanol) and paraffin oil (100 µl to prevent urine evaporation). Samples were frozen at −70°C until analysis. All sugars were quantified by ion exchange high-performance liquid chromatography (HPLC) as previously described.^23^ Briefly, cellobiose was added as an internal standard, and the urine was filtered through a 0.4 µm filter and diluted as necessary. Samples were deionised and then injected on a Dionex MA-1 ion exchange column (Dionex, Sunnyvale, California, USA). Sugars were eluted with NaOH at a flow rate of 0.4 ml/min. Peaks were detected using pulsed amperometric detection on a Dionex HPLC and quantified as peak areas. Final data were reported as either fractional excretions (sucrose and sucralose) or as a ratio of fractional excretions (lactulose/mannitol). Fractional excretion is defined as the fraction of the gavaged dose recovered in the urine sample.

Sucralose was also assayed by HPLC. Separation was achieved using a Dionex Ionpac NS1 column and acetonitrile–water as the eluent at a flow rate of 1 ml/min. Detection was performed with an electrochemical detector in a fashion identical to the other sugars. For these assays, the internal standard used was phenyl-β-d-thiogalactoside (Sigma Chemical, Oakville, Ontario, Canada) added to the initial urine sample at a concentration of 0.1 mg/ml.

### Mucosal cytokine secretion

At 4, 8 and 17 weeks of age, colonic organ cultures were prepared from control mice, IL10^−^/^−^ mice, and IL10^−^/^−^ mice treated with AT-1001. Because of the patchy nature of colitis in IL10^−^/^−^ mice whole colons were removed, flushed with PBS, and a quarter of the tissue was cut longitudinally and resuspended in tissue culture plates (Falcon 3046; Becton Dickinson Labware, Lincoln Park, New Jersey, USA) in RPMI 1640 supplemented with 10% fetal calf serum, penicillin (100 U/ml), streptomycin (100 U/ml). Cultures were incubated at 37°C in 5% CO_2_. After 24 h, supernatants were harvested and stored at −70°C for analysis of cytokine levels. Pellets were left to dry for 4 days and weighed. TNFα and IFNγ levels in cell supernatants were measured using enzyme-linked immunosorbent assay (ELISA) kits (Medicorp, Montreal, Quebec, Canada).

### Myeloperoxidase assay

At 17 weeks of age, a section of the colon of all animals was removed upon dissection, weighed and immediately flash-frozen. Samples were kept at −70°C until determination of myeloperoxidase (MPO) concentrations. Levels of MPO in colonic tissue were measured using an ELISA kit (Cell Sciences, Canton, Massachusetts, USA).

### Histological injury grading

Upon sacrifice, proximal, mid, distal ileum and whole colon were harvested and fixed in 10% phosphate-buffered formalin. These samples were embedded in paraffin in toto, sectioned at 4 μm, and stained with haematoxylin & eosin for light microscopy examination. The slides were reviewed in a blinded fashion by a pathologist (JSD) and were assigned a histological score for intestinal inflammation using a scheme modified from Saverymuttu *et al*.^24^

### Statistical analysis

Data are expressed as means (SEM). Comparisons between groups were made using ANOVA with a Tukey test for post hoc comparisons. All calculations were performed using Graphpad Prism 3.0, and significance was assumed at p<0.05.

## RESULTS

### Permeability

**Figure 1:**
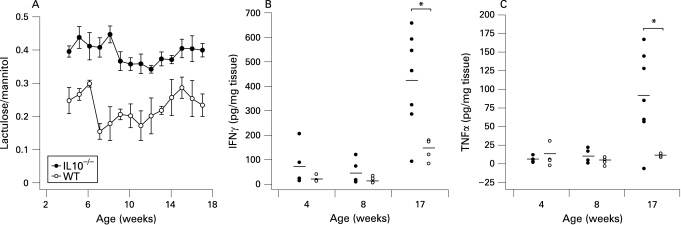
Increased small intestinal permeability precedes colonic disease. (A) small intestinal permeability measurements from 4 to 17 weeks in interleukin 10 gene-deficient (IL10^−^/^−^) mice (closed circles) and Sv/Ev 129 wild-type mice (open circles) show a marked difference between these animal models very early in age. (n = 10–13 at each time point). (B) Concentration of interferon γ (IFNγ) in the small intestine of IL10^−^/^−^ (closed circles) and wild-type mice (open circles) at 4, 8 and 17 weeks of age. (C) Concentration of tumour necrosis factor α (TNFα) in the small intestine of IL10^−^/^−^ (closed circles) and wild-type mice (open circles) at 4, 8 and 17 weeks of age. For both cytokines, a significant difference was observed between the IL10^−^/^−^ and wild-type mice only at 17 weeks. (p<0.001; n = 4–7).

To determine whether there was a primary permeability defect in IL10^−^/^−^ mice, we examined small intestinal permeability from 4 to 17 weeks of age and compared it to wild-type mice. The IL10^−^/^−^ mice showed an increased small intestinal permeability from 4 weeks onwards compared to the Sv/Ev 129 wild-type mice (fig 1A). The proinflammatory cytokines IFNγ and TNFα have been shown to be elevated in the colon of this animal in the presence of colitis.^18^ In order to establish whether the small intestinal permeability defect preceded colitis, we measured the secretion of these cytokines in the colon at 4, 8 and 17 weeks of age. As shown in fig 1B,C the IL10^−^/^−^ and wild-type mice produced similar levels of IFNγ and TNFα at 4 and 8 weeks but their cytokine secretion levels were significantly greater at 17 weeks of age, when the animals exhibited colitis. Clearly, abnormal small intestinal permeability preceded the development of colitis by this criterion.

**Figure 2:**
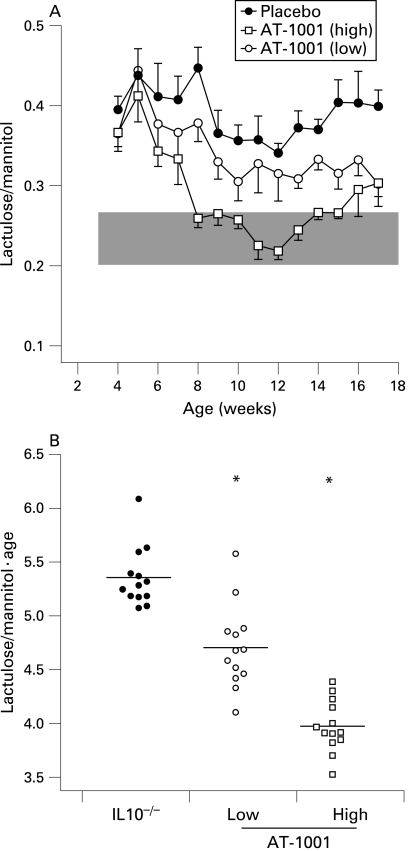
Small intestinal permeability is reduced with a zonulin antagonist AT-1001. (A) Small intestinal permeability was measured in IL10^−^/^−^ mice treated with high dose AT-1001 (open squares), low dose (open circles) or placebo (closed circles) from 4 to 17 weeks of age. The shaded area represents the mean (3 SD) of the lactulose/mannitol ratios observed in wild-type mice. Mice treated with the high dose of AT-1001 eventually reached the wild-type range. (B) Statistical analysis of the areas under the curve of small intestinal permeabilities for each group. The permeabilities of the groups treated with AT-1001 differed significantly from the placebo group; p<0.05 (low dose), p<0.01 (high dose), n = 10–13.

To determine whether the increased small intestinal permeability was zonulin dependent the animals were treated with AT-1001. When given daily in the drinking water the zonulin antagonist AT-1001 was effective in decreasing small intestinal permeability in the IL10^−^/^−^ mice (fig 2). After 4 weeks of treatment, high-dose AT-1001 reduced small intestinal permeability to levels observed in control animals. This was evident from 8 weeks of age (fig 2A). The animals treated with low dose AT-1001 showed a significant reduction in overall permeability (as expressed as the area under the permeability curve) as compared to the placebo group (fig 2B), but this dose did not quite return small intestinal permeability to normal levels (fig 2A).

**Figure 7:**
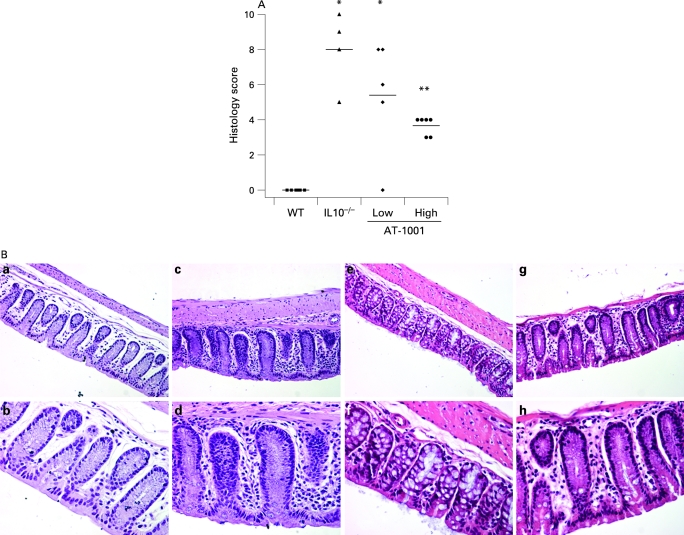
(A) Histological scoring. The interleukin 10 gene-deficient (IL10^−/−^) mice and the animals treated with a low dose of the zonulin peptide inhibitor AT-1001 had significantly increased histological scores as compared to the wild-type mice (*p<0.05). However, the group treated with a high dose of AT-1001 had a histological score significantly lower than the untreated IL10^−/−^ animals (**p<0.05), but this was still elevated significantly from the wild-type (WT) controls (n = 4–6). (B) Representative histology. All panels show colonic histology at 17 weeks of age. The upper panels (a, c, e, g) were taken at ×20 magnification, while the lower panels (b, d, f, h) are the same regions at ×40. Panels a/b represent tissue from the wild-type animals while panels c/d are from the IL10^−^/^−^ mice. A clear inflammatory infiltrate is observed in the lamina propria of these animals. Panels e/f are from the low-dose group while g/h are from the high-dose treated animals. It can be appreciated that there is a significant reduction in the inflammatory component of the lesion observed in these animals.

A similar attenuation effect was observed histologically. These results are illustrated in fig 7. Significant inflammation was observed in the IL10^−^/^−^ mice which was similar to that observed in those treated with the low dose of AT-1001. However, those animals treated with the high dose of the compound had a significant reduction in this histological score, although it was still significantly greater than the scores observed in the control animals.

**Figure 3:**
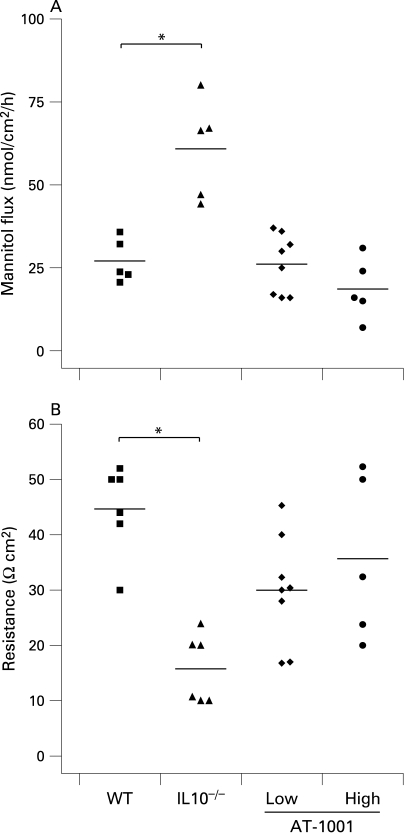
The zonulin peptide inhibitor AT-1001 reduced small intestinal permeability by 8 weeks. (A) Mannitol flux was measured in Ussing chambers at 8 weeks of age and was increased in the interleukin 10 gene-deficient (IL10^−^/^−^) mice (p<0.05). This increase was not observed in animals treated with AT-1001 (n = 4–8). (B) The electrical resistance was reduced in the IL10^−/−^ mice only (p<0.01). AT-1001 treatment prevented this reduction (n = 5–8). WT, wild-type mice.

The effect of AT-1001 in reducing small intestinal permeability was also evident in vitro at 8 weeks of age. As demonstrated in fig 3, small intestinal permeability to mannitol and electrical resistance were determined in Ussing chambers. Both doses of AT-1001 effectively reduced permeability to mannitol in the small intestine (fig 3A). Furthermore, the loss of electrical resistance, observed in the IL10^−^/^−^ animals, was prevented in the high dose treated group, but not in the low dose treated group (fig 3B).

### Inflammation

To test whether or not decreasing small intestinal permeability would affect the development of colonic disease, we evaluated several parameters of colonic disease. Over the entire course of the experiment we non-invasively determined epithelial colonic damage using the clearance of orally administered sucralose. Additional markers of colitis included in vitro colonic permeability at 8 weeks of age, and at 17 weeks of age, colonic cytokine secretion, MPO content and histology.

**Figure 4:**
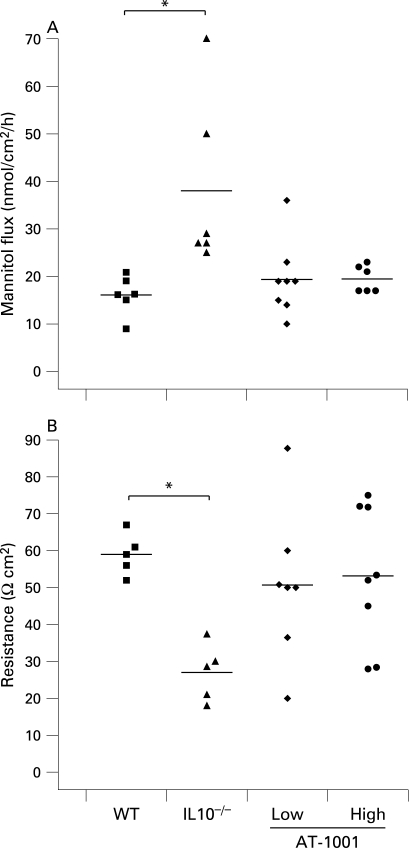
Attenuation of colonic permeability at 8 weeks. (A) Colonic mannitol flux was increased in the interleukin 10 gene-deficient (IL10^−/−^) mice (p<0.05). This increase was prevented by the zonulin peptide inhibitor AT-1001 (n = 5–8). (B) Electrical resistance was reduced in the IL10^−/−^ mice and again prevented by AT-1001 (p<0.05, n = 5–8). WT, wild-type mice.

At 8 weeks of age colonic permeability to mannitol was increased in the IL10^−^/^−^ animals with a corresponding decrease in electrical resistance. In the treated animals these changes were largely prevented and both treated groups showed similar results to wild-type mice (fig 4A,B). This suggests that the early evidence of colonic disease, observed in IL10^−^/^−^ mice, is prevented or delayed by treatment with AT-1001.

**Figure 5:**
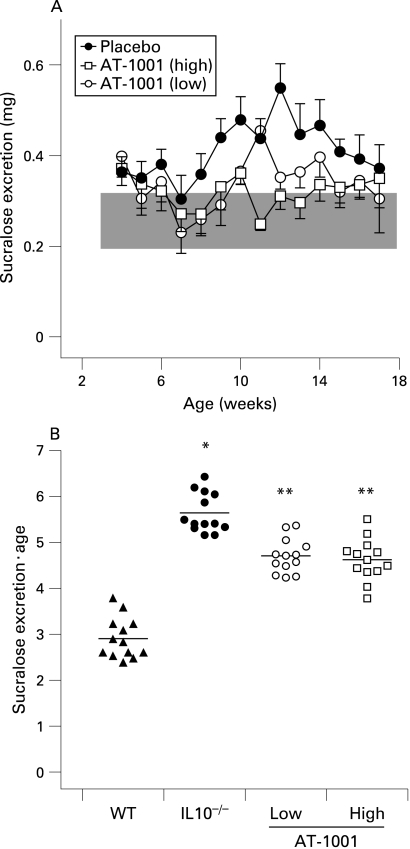
The zonulin peptide inhibitor AT-1001 attenuates colonic damage. (A) Sucralose excretion was measured weekly in interleukin 10 gene-deficient (IL10^−^/^−^) mice treated with a high dose of AT-1001 (open squares), low dose (open circles) or placebo (closed circles) from 4 to 17 weeks of age. The data are shown relative to that from wild-type (WT) animals, which is represented in the shaded area. As noted in the text colonic damage becomes evident at 10 weeks of age but is reduced in AT-1001 animals. (B) Statistical analysis of the areas under the curve of sucralose excretion for each group. The sucralose excretion of IL10^−/−^ mice was significantly higher than that from wild-type animals (*p<0.01). Sucralose excretion of the animals treated with AT-1001 differed significantly from that of the IL10^−/−^ mice (**p<0.05, n = 10–13), suggesting that AT-1001 reduces but does not prevent colonic damage.

Colonic damage was measured weekly using sucralose excretion (fig 5). IL10^−^/^−^ mice and controls excreted similar amounts of sucralose in their urine until week 10, following which they started showing elevated amounts of sucralose (fig 5A). IL10^−^/^−^ animals had a significantly increased cumulative amount of damage as compared to the wild-type animals (fig 5B). Both treated groups had significantly less damage than the IL10^−^/^−^ animals but still significantly more than the wild-type group. Treatment with AT-1001 appears to have been significantly ameliorated, but not completely prevented colonic disease as determined by this parameter.

**Figure 6:**
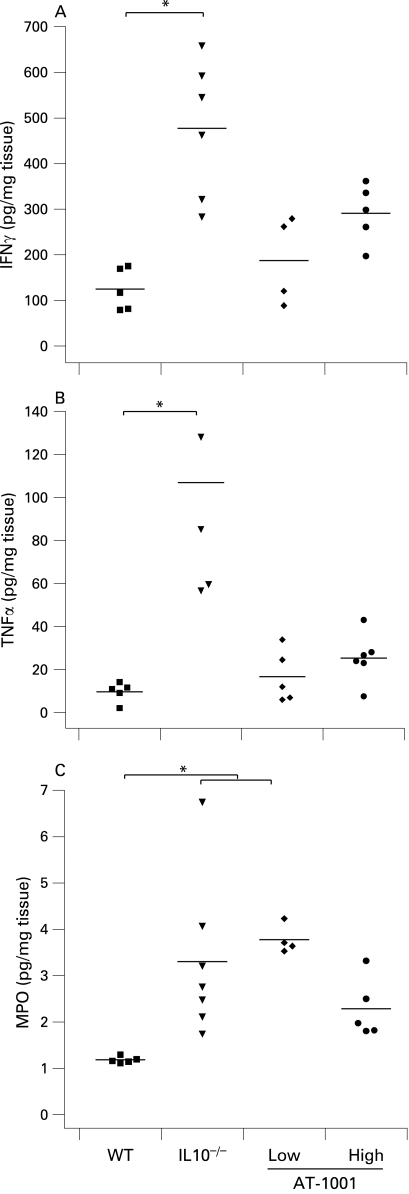
Colonic secretion of interferon γ (IFNγ), tumour necrosis factor α (TNFα) and myeloperoxidase (MPO) content at 17 weeks. (A and B) The interleukin 10 gene-deficient (IL10^−/−^) mice had a significant increase in colonic secretion of these cytokines (p<0.05). This increase was prevented by treatment with the zonulin peptide inhibitor AT-1001 (n = 4–6). (C) The IL10^−/−^ mice and the mice treated with the low dose had a significant increase in MPO content (p<0.05). High-dose treatment of AT-1001 prevented this increase (n = 4–6).

At 17 weeks of age, IFNγ and TNFα secretion in the colon was clearly reduced in the groups treated with AT-1001. Only the IL10^−^/^−^ group showed significantly increased rates of cytokine secretion compared to the wild-type animals (fig 6A,B). Neutrophil infiltration in the colon, as measured by tissue MPO content (fig 6C), was increased in the IL10^−^/^−^ and low dose AT-1001 mice as compared to controls but was not significantly increased in the high dose treated group. Taken together, these data suggest that treatment with AT-1001, active in the small intestine was effective at reducing the colonic inflammation observed in these animals.

Representative examples of the colonic histology are presented in fig 7. The upper panels are taken at ×20 magnification whilst the lower are at ×40. In fig 7B, panels a/b illustrate the histological features observed in the background strain of animals with only a sprinkling of inflammatory cells in the lamina propria. This is in contrast to the IL10^−^/^−^ mice shown in panels c/d where there is a marked increase in the inflammatory cell compartment. Panels e/f and g/h are representative sections taken from animals treated with the low and high doses of AT-1001, respectively. It can be appreciated that the inflammatory changes observed in these animals are less than those seen in the IL10^−^/^−^ mice.

**Figure 8:**
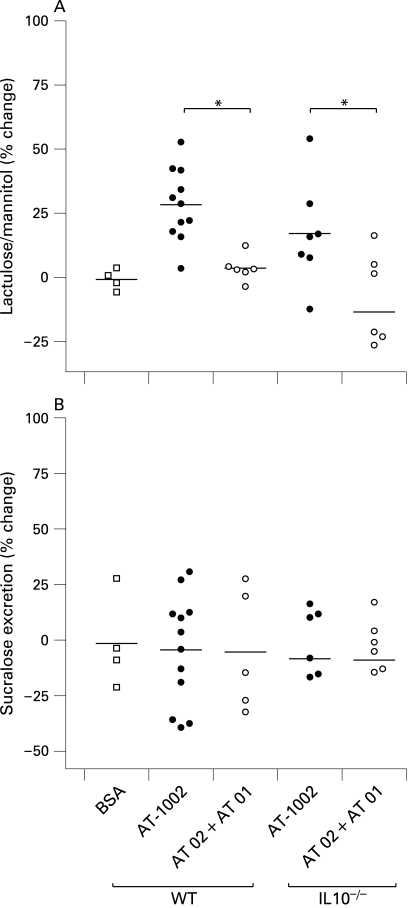
Zonulin pathway mediated changes in permeability. (A) Mice were treated with the zonulin peptide agonist AT-1002 (1 mg/ml) or a mixture of AT-1002 and the zonulin peptide inhibitor AT-1001 (1 mg/ml each) for 24 h. The zonulin agonist increased small intestinal permeability (lactulose/mannitol ratio). The concomitant administration of the agonist (AT 01 + AT 02) abrogated this increase. The same effect was demonstrated in IL10^−/−^ animals. Bovine serum albumin (BSA) was used as a protein control (p<0.05; n = 4–12). (B) Colonic permeability (sucralose excretion) was determined in the same animals. In contrast to small intestinal permeability, stimulation of the zonulin pathway had no effect in the colon of either wild-type or IL10^−/−^ mice (4–12).

It has been suggested that the zonulin receptor is absent in the colon; however, that work was done in rabbits, not mice.^16^ Therefore, to rule out the possibility that this pathway is present in the colon and that the effects observed throughout our experiments were a consequence of the drug in the colon, we performed a functional assay to test for the presence of a zonulin effect in the colon using a zonulin peptide agonist (AT-1002). As observed in fig 8, after challenge with this agent, only small intestinal permeability was increased, while sucralose excretion stayed the same. This was true for both the IL10^−/−^ and wild-type mice. Using increasing concentrations of AT-1002 we were able to demonstrate that the increase in the lactulose/mannitol ratio was dose dependent and at no dose, up to the solubility limit of AT-1002, did we observe any effect in the colon (data not shown). When both wild-type and IL10^−/−^ animals were treated with a combination of AT-1001 and AT-1002, the increase in small intestinal permeability was abolished, demonstrating that the effect of AT-1002 to increase small intestinal permeability could be prevented or blocked by the same agent we used in the previous experiments.

## DISCUSSION

The aetiology of Crohn’s disease is poorly understood. However, it has been proposed that three main features must be present for disease to occur: (1) a genetically susceptible mucosal immune system; (2) an antigen, or pro-inflammatory compound, which reaches the gut and can trigger the susceptible immune system; and (3) an alteration in gut barrier function which allows this antigen to have contact with the mucosal immune system.^19^ In this regard the IL10 deficient mouse is an interesting model of human Crohn’s colitis. It does not develop colitis in the absence of intestinal bacterial flora, suggesting that one or more components within the enormously complex flora trigger the susceptible mucosal immune system to develop inflammation.

The experiments presented here provide evidence that a break in small intestinal barrier function is necessary for colitis to occur in the IL10^−^/^−^ mouse. This and other animal models of Crohn’s disease, including the senescence accelerated prone mouse (SAMP) and the mouse downregulated in adenoma (mdra) deficient mouse have shown increased small intestinal permeability well before disease expression.^25^^–^27 This study extends these observations in an important manner by demonstrating that reversal of this barrier defect can attenuate the disease, implying that the increased permeability is not simply an epiphenomenon but rather is an important aetiological event.

Our in vivo measurements of small intestinal permeability indicated that these mice demonstrate abnormal small intestinal permeability at 4 weeks of age, which is as early a time point as we can measure. In fact it has been previously demonstrated that this defect can be detected using ex vivo techniques at 2 weeks of age, in the absence of histological injury.^25^ In this study we measured the secretion of proinflammatory cytokines as a marker of disease,^25^ ^28^ and by this criteria demonstrated that increased small intestinal permeability preceded disease.

Normal gastrointestinal permeability is tightly regulated and the mechanisms involved are, for the most part, unknown. However, recent work suggests that one biological pathway that regulates permeability involves zonulin. This hormone appears to be secreted into the lumen and activates an apical receptor which initiates a cascade of events culminating in the opening of the tight junction. The identification of this pathway has also provided an opportunity for pharmacological manipulation of increased permeability associated with over-activity of this pathway. Because zonulin receptors are not present in colonic epithelium,^16^ drugs that work via this mechanism should not have effects in the colon. To confirm that this was true in our mice, we tested whether a zonulin-mediated event could be elicited in the colon under conditions identical to those used in our experiments. We used AT-1002, a peptide very similar to AT-1001 but an agonist of the zonulin receptor, and found that at a high concentration it could only increase small intestinal permeability. Moreover, when both the agonist and antagonist were given together the change in small intestinal permeability was prevented, suggesting that they act through the same pathway. These data suggest that the zonulin receptor is not present in the colon of mice (as described in the rabbit) but we cannot exclude the possibility that the peptides were simply degraded during their transit of the small intestine. However, regardless of which explanation is correct, these data suggest that the attenuation of colitis observed in this study following treatment with AT-1001 was due to its effect in the small intestine.

It is important to recognise that in our experiment even though colitis was ameliorated it was not abolished. Even in the treated animals there was still histological evidence of mild inflammation as well as MPO accumulation. There are several potential explanations for this. First, animals received treatment only from the age of 4 weeks and small intestinal permeability was not reduced to “normal” until 8 weeks of age. Since the animals are weaned from their mothers at 3–4 weeks of age it is difficult to initiate this therapy any earlier. However, we know that the permeability defect is present by 2 weeks of age and this means that we did not completely remove this potential inflammatory stimulus. Second, it is entirely possible that the colonic disease in this animal model is not only generated by events that occur proximally in the small intestine, but also by direct interaction of the colonic bacterial flora with the IL10 deficient immune system. Thus, reducing antigen presentation in the small intestine may not completely abrogate disease as bacterial components could still induce secretion of proinflammatory cytokines in the colon and perpetuate inflammation.^8^ ^29^

In conclusion, we believe that these experimental data support the hypothesis that, in a genetically predisposed host, increased small intestinal permeability can contribute to the induction of inflammatory disease. In the BB rat the disease is expressed in the pancreatic islet cells,^20^ and reducing the abnormal permeability that precedes this disease with AT-1001 can ameliorate the resulting diabetes.^21^ The current study has extended these observations by demonstrating that in the IL10^−^/^−^ mouse abnormal small intestinal permeability not only precedes the development of colitis but it is also aetiologically important. Reducing small intestinal permeability with AT-1001 significantly attenuates colitis.
